# Applying machine learning to predict future adherence to physical activity programs

**DOI:** 10.1186/s12911-019-0890-0

**Published:** 2019-08-22

**Authors:** Mo Zhou, Yoshimi Fukuoka, Ken Goldberg, Eric Vittinghoff, Anil Aswani

**Affiliations:** 1 Department of Industrial Engineering and Operations Research, University of California at Berkeley, 4141 Etcheverry Hall, Berkeley, CA 94720 USA; 2 Department of Physiological Nursing, School of Nursing, University of California at San Francisco, 2 Koret Way, N631, San Francisco, 94143 USA; 3 Department of Industrial Engineering and Operations Research & Electrical Engineering and Computer Sciences, University of California at Berkeley, 425 Sutardja Dai Hall, Berkeley, CA 94720-1777 USA; 4 Department of Epidemiology & Biostatistics, School of Medicine, University of California at San Francisco, 550 16th. Street, San Francisco, CA 94158 USA; 5 Department of Industrial Engineering and Operations Research, University of California at Berkeley, 4119 Etcheverry Hall, Berkeley, CA 94720-1777 USA

**Keywords:** Physical activity, Exercise relapse, Adherence, Machine learning

## Abstract

**Background:**

Identifying individuals who are unlikely to adhere to a physical exercise regime has potential to improve physical activity interventions. The aim of this paper is to develop and test adherence prediction models using objectively measured physical activity data in the Mobile Phone-Based Physical Activity Education program (mPED) trial. To the best of our knowledge, this is the first to apply Machine Learning methods to predict exercise relapse using accelerometer-recorded physical activity data.

**Methods:**

We use logistic regression and support vector machine methods to design two versions of a Discontinuation Prediction Score (DiPS), which uses objectively measured past data (e.g., steps and goal achievement) to provide a numerical quantity indicating the likelihood of exercise relapse in the upcoming week. The respective prediction accuracy of these two versions of DiPS are compared, and then numerical simulation is performed to explore the potential of using DiPS to selectively allocate financial incentives to participants to encourage them to increase physical activity.

**Results:**

we had access to a physical activity trial data that were continuously collected every 60 sec every day for 9 months in 210 participants. By using the first 15 weeks of data as training and test on weeks 16–30, we show that both versions of DiPS have a test AUC of 0.9 with high sensitivity and specificity in predicting the probability of exercise adherence. Simulation results assuming different intervention regimes suggest the potential benefit of using DiPS as a score to allocate resources in physical activity intervention programs in reducing costs over other allocation schemes.

**Conclusions:**

DiPS is capable of making accurate and robust predictions for future weeks. The most predictive features are steps and physical activity intensity. Furthermore, the use of DiPS scores can be a promising approach to determine when or if to provide just-in-time messages and step goal adjustments to improve compliance. Further studies on the use of DiPS in the design of physical activity promotion programs are warranted.

**Trial registration:**

ClinicalTrials.gov NCT01280812 Registered on January 21, 2011.

## Background

Despite the health benefits of physical activity, adherence to physical activity programs can be challenging. Low adherence to a prescribed physical activity regime can significantly diminish the short- and long-term benefits of such programs. Factors that are associated with adherence are barriers for activity, self-efficacy, exercise history, health condition, stress, social support, physical environment, and cognitive activities [1–4]. Early prediction of individuals who are likely to relapse can significantly improve adherence to physical activity interventions. However, continuously measured objective physical activity data are rarely available to researchers to develop such prediction models. For example, the NHANES 2003–2004 and 2005–2006 studies include only 7 consecutive days of accelerometer-measured data in 9601 adults and 5030 children [5, 6]. Fortunately, we had an opportunity to access to the physical activity trial data that were continuously collected every 60 sec every day for 9 months in 210 participants [7].

Adherence can vary on a day-to-day basis with even usually-adherent individuals having temporary relapses [8]. Inadequate skills and knowledge for high-risk situations are a leading factor in temporary relapse. Such relapse often results in an “abstinence violation effect” that leads to a perceived loss of control and eventually total relapse [9–11]. Much work on predicting adherence has focused on the use of sociodemographics and self-reported questionnaire data [12–14]. However, real-time data collection such as from electronic health records (EHR), wearable devices, or mobile phones can potentially increase the prediction accuracy of adherence. One approach [15] used EHR data to construct a Markov chain model to predict medication adherence, where the model states were frequency of taking medication. Unfortunately, this kind of model is not applicable to personalized interventions where adherence is measured relative to a baseline that varies for each individual. Another approach [16] used mobile data to construct a utility-function model of behavior in weight loss programs. Though the model is personalized to the baseline physical activity of each individual, this model predicts future physical activity and not adherence. Given the potential value of predicting adherence to medical treatments using EHR and mobile data, there is a need for the development and validation of new models that can make such predictions.

### Review of Mobile technologies and physical activity

Physical activity promotion programs can benefit from adherence predictions. The 2018 National Physical Activity Guidelines for American recommend that adults engage in at least 150 min to 300 min a week of moderate-intensity, or 75 min to 150 min a week of vigorous-intensity aerobic physical activity, or an equivalent combination of moderate- and vigorous-intensity aerobic activity [17]. However, objectively measured physical activity data indicated that only a small proportion of American adults met the guidelines [18]. Furthermore, physical activity programs suffer from exercise relapses and low adherence, which hinders individuals from meeting the guidelines. Despite the potential of leveraging mobile technologies with activity trackers and wearable devices to provide accurate real-time measurements of physical activity and deliver interventions to encourage adherence, [19–22] the capacity of these technologies in automating and personalizing physical activity promotion programs is only beginning to be explored. Recent studies have found that mobile-based lifestyle modification programs with a reduced number of coaching sessions can achieve statistically significant increases in physical activity [23–34]. Encouraged by this success, a more recent question is whether it is feasible to use mobile technologies to deliver fully-automated or nearly-fully-automated physical activity promotion programs. One study [23] sent personalized messages based on self-reported assessments, but did not use objectively measured data for personalization. Our prior study, a fully-automated physical activity intervention with a personalized goal setting feature, validated the feasibility of adopting additional levels of automation to improve the efficacy of such programs in a cost-effective way [35]. Full automation can enable further scaling of these programs to larger populations and accurate prediction of adherence is important.

### Review of warning scoring in healthcare

Several warning scores have been developed in healthcare for the purpose of predicting adverse medical events at an early stage so that medical interventions can be delivered before significant patient state deterioration occurs. Early Warning Scoring (EWS) is a popular system for bedside patient assessment [36–38]. It is based on the physiologic assessment of multiple vital signs (e.g., respiration, heart rate, body temperature, etc.) and abnormal observations, which trigger immediate notifications that lead to early interventions to prevent critical events from happening. Validated EWS algorithms are also used to provide guidance for optimizing patient management and guiding resource allocation within healthcare organizations [39, 40].

Despite the wide adoption of EWS systems in critical care medicine, similar types of warning scores have not been developed for or applied to physical activity interventions. Since exercise relapse tends to hinder further participation in physical activity, an early warning scoring system with accurate predictions on exercise relapse can be used to guide the provision of immediate interventions and has the potential to increase adherence.

### Aims

In this paper, we use logistic regression (LR) and support vector machine (SVM) methods to design two versions of a Discontinuation Prediction Score (DiPS), which uses each individual’s past data (e.g., physical activity duration, physical activity intensity and goal achievement) to assign a numeric value that quantifies their likelihood of discontinuing physical activity in the upcoming week. The potential utility of DiPS is to provide guidance for provision of just-in-time interventions for individuals who are more likely to have an exercise relapse. We conducted a simulation to compare the cost-effectiveness of a DiPS-based policy in promoting adherence. To the best of our knowledge, this is the first to apply Machine Learning methods to predict exercise relapse using accelerometer-recorded physical activity data.

This paper is organized as follows: We first describe the dataset of the Mobile Phone-Based Physical Activity Education program (mPED). Then we describe our *feature engineering* procedure, where the raw data for each individual is converted into a set of summary statistics for each individual. Next, we define two versions of DiPS using logistic regression and SVM, and quantify the prediction accuracy of DiPS using an out-of-sample evaluation methodology. Lastly, we discuss how DiPS can be integrated into physical activity promotion programs and present a simulation to explore the potential benefit of using DiPS-based interventions to increase adherence in a cost-effective way.

## Methods

### Study design and data description

This is a secondary data analysis of the mPED study which is a randomized controlled trial (RCT). The main results of the trial were published in [7]. The study protocol was approved by the University of California, San Francisco Committee on Human Research (CHR) and the mPED Data and Safety Monitoring Board.

#### The mPED dataset

In this paper, we used the data of 210 community dwelling physically inactive women, age 25 to 69 years. In brief, this mPED trial was an unblinded, parallel randomized controlled trial (RCT) conducted with three groups (CONTROL, REGULA, and PLUS groups). The trial consisted of a 3-week run-in period, a 3-month intervention period using the app, accelerometer, and brief counseling to increase physical activity, and a 6-month maintenance period using accelerometer (and the app) to maintain activity. The run-in period was conducted to collect average baseline physical activity (daily steps and MVPA). The CONTROL group was asked to use an accelerometer for the entire 9-month (36-week) study period, but did not receive any physical activity intervention. In contrast, the REGULAR and PLUS groups received the identical physical activity intervention, consisting of accelerometer, brief in-person counseling sessions, and mPED trial app for the first 3 months (12 weeks). Once registering each participant’s average baseline daily steps onto the mPED trial app, the app started displaying her weekly daily step goals which were set to increase at a 20% rate from her average baseline daily steps. Once her daily step goals reached 10,000 steps, she was asked to maintain at least 10,000 steps per day, 7 days a week during the remaining study period. Personalized automated feedback was provided daily via the mPED trial app. In the 6-month (24-weeks) maintenance period, the PLUS group kept both the mPED trial app and accelerometer, while the REGULAR group kept using only the accelerometer. The overall participant retention rate was 97.6% at 9 months (36 weeks)[7]. In the mPED trial, physical activity was measured using a triaxial accelerometer (HJA-350IT, Active style Pro, Omron Healthcare Co., Ltd.). This accelerometer has been previously validated [41, 42]. The accelerometer automatically reset the count each midnight,and allowed participants to view their counts for the past 7 days during the 3-month intervention and 6-month maintenance period. They were instructed to place the accelerometer on the waist in line with the middle of the thigh of their dominant leg and wear it from the time they got up in the morning until they went to bed at night every day except when showering, bathing, swimming, or sleeping at night. Activity data from the most recent 150 days were automatically stored and directly downloaded to a computer in our research office. The following types of physical activity data were collected:

### METs data

The mean intensity value of a 1-min epoch is calculated as the average value of six 10s epochs. Based on the METs recordings, physical activity is automatically classified as no measurement, lifestyle activity and walking activity. Moderate to vigorous intensity physical activity (MVPA) is METs ≥3.

### Steps data

The accelerometer provides information on the steps value of a 1-h epoch and daily steps.

#### Features

We extracted a set of interpretable features from the objectively measured physical activity data. For each participant, we defined the following features:

1. Week number *t*: the number of weeks in the study.
2. Average daily steps: average of daily steps from the first day of the run-in period to the last day of week *t* − 1.
3. Initial average daily steps: average of daily steps in the run-in period.
4. Last week average daily steps: average of daily steps in week *t* − 1.
5. Average goal achieving percentage: percentage of step goals achieved from the first day of the run-in period to the last day of week *t* − 1.
6. Last week goal achieving percentage: percentage of step goals achieved in week *t* − 1.
7. Average MVPA minutes in the morning: average number of minutes with METs ≥3 in the morning (3:00–9:59) from the first day of the run-in period to the last day of week *t* − 1.
8. Initial MVPA minutes in the morning: average number of minutes with METs ≥3 in the morning (3:00–9:59) in the run-in period.
9. Last week MVPA minutes in the morning: average number of minutes with METs ≥3 in the morning (3:00–9:59) in week *t* − 1.
10. Average MVPA minutes in the afternoon: average number of minutes with METs ≥3 in the afternoon (10:00–14:59) from the first day of the run-in period to the last day of week *t* − 1.
11. Initial MVPA minutes in the afternoon: the average number of minutes with METs ≥3 in the afternoon (10:00–14:59) in the run-in period.
12. Last week MVPA minutes in the afternoon: average number of minutes with METs ≥3 in the afternoon (10:00–14:59) in week *t* − 1.
13. Average MVPA minutes in the evening: average number of minutes with METs ≥3 in the evening (15:00–3:00) from the first day of the run-in period to the last day of week *t* − 1.
14. Initial MVPA minutes in the evening: average number of minutes with METs ≥3 in the evening (15:00–3:00) in the run-in period.
15. Last week MVPA minutes in the evening: average number of minutes with METs ≥3 in the evening (15:00–3:00) in week *t* − 1.
16. Average MVPA intensity: average METs readings for METs ≥3 from the first day of the run-in period to the last day of week *t* − 1.
17. Initial MVPA intensity: average METs readings for METs ≥3 in the run-in period.
18. Last week MVPA intensity: the average METs readings for METs ≥3 in week *t* − 1.

Daily steps reflect the participant’s overall daily physical activity. Goal-achieving percentage demonstrates the participant’s response to step goals. MVPA in different time in day expresses the preferred time in day of performing MVPA, and MVPA intensity is coarsely indicative of the type of physical activity performed. We separated a day into three intervals: morning (3:00–9:59), afternoon (10:00–14:59), and evening (15:00–3:00) because prior clustering analysis on this dataset [43] identified three clusters of individuals who tend to do physical activity in the morning (3:00–9:59), afternoon (10:00–14:59), and evening (15:00–3:00), respectively.

Restated, our defined set of features includes daily steps, goal-achieving percentage, MVPA in the morning, MVPA in the afternoon, MVPA in the evening and MVPA intensity: The complete set of features includes all 18 features listed above, where we included the set of features for the run-in period, in the last week and over the entire study period. The set of features for the run-in period are included to account for the initial differences between participants. The features on last week behavior capture the immediate past performance. The features on average behavior demonstrate the overall performance of the participant so far during the study. The week number is included to model the changes in behavior over time.

### Analytical methods

In this section, we first define the Discontinuation Prediction Score (DiPS) in the context of a clinical trial. Then we move on to introduce the statistical models (i.e., logistic regression and Support Vector Machine) used to develop this score.

#### DiPS definition

DiPS aims to predict the probability of having exercise relapse (binary) for a particular participant at each week of a physical activity promotion program based on recorded physical activity data in the early weeks. DiPS outputs a score (between 0 and 1) to suggest how likely is the participant to have an exercise relapse for the following week. For this paper, we use [0,1] as the interval for DiPS since the model output is a probability. We further define that a participant is having an exercise relapse in a given week if the average steps in that week is lower than the average steps in the run-in period. The reason we use the run-in period average is that the mPED trial was designed so that true baseline steps data is collected from each participant during the run-in period. Since the aim of these programs is to increase participants’ daily steps, comparison with the run-in average serves as a useful signal reflecting the progress of the participant.

Recall that the 2018 National Physical Activity Guidelines use a week as the unit to measure activity time for different physical activity intensity [17]. We adopt a similar approach to use a week as an assessment unit. Furthermore, this granularity can mitigate the impact of large day-to-day fluctuations of daily steps. Note that for a particular week in the past (thus with known data), a participant has a DiPS of 0 if his/her weekly average step is lower than his/her run-in average steps and a DiPS of 1 if his/her weekly average steps is higher than his/her run-in average steps. Therefore in the training phase, DiPS can be regarded as a binary variable (since it is either 0 or 1). But in the prediction phase, DiPS is a continuous variable in the range of [0,1], indicating the likelihood of achieving an above run-in step in the following week.

#### Pre-processing

**Fig. 1:**
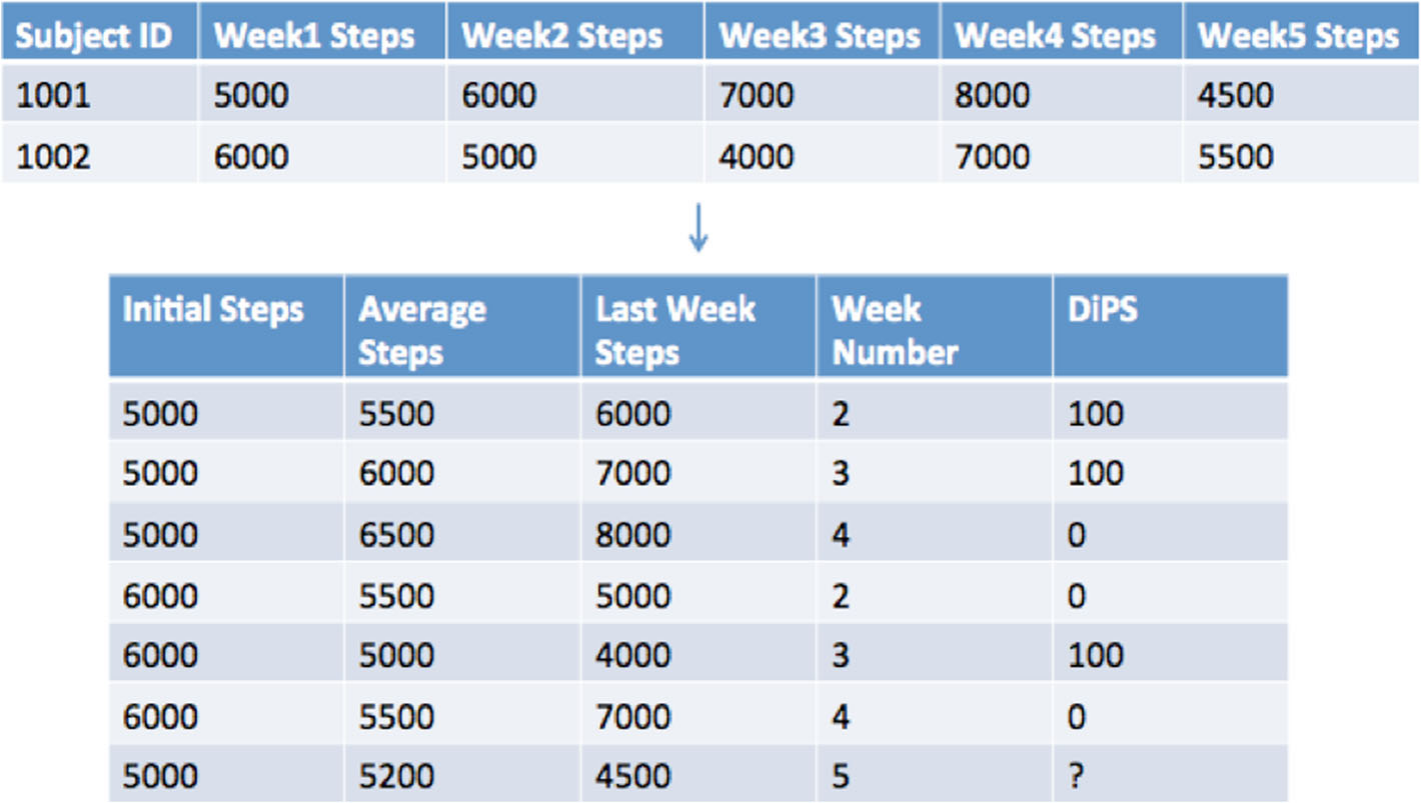
Simplified example of training data augmentation. Caption: the first table shows the raw physical activity data for two different participants 1001 and 1002 before augmentation and the second table shows the resulting data after augmentation, where the first six rows are the training data and the last row is the testing data

Recall that the mPED dataset contains 210 participants. If we regard each participant as a single data point, our sample is too small for the model to learn. Therefore, we first augmented the training data by assuming that the relationship between week ***i*** and ***i +*** **1** is independent of the relationship between week ***j*** and ***j +*** **1** for ***i ≠ j***, and under this assumption, we can augment the training data to include features for each participant for each observed week. For instance, assume we are at week ***n*** of the study and we want to train the model, then instead of using a single observation for each participant using data in week ***n −*** **1** as the response variable, our new approach creates a set of observations for the participant, where each observation uses data in week 3*,* 4*, ..,* ***n −*** **1** as the response variable and the corresponding features are from weeks prior to that week. For example, suppose we are at the end of week 5 of the study and would like to generate observations for participant 1001. Then the augmented training data contains 3 observations for participant 1001, i.e., the complete set of features for week 2, 3 and 4; and the response variable is the observed DiPS of participant 1001 in week 3, 4 and 5 respectively, i.e., 0 if the participant’s average step in that week is below the participant’s average step in the run-in period, 1 otherwise. Figure 1 illustrates a simplified example of this training data augmentation procedure.

### Statistical models

We developed two versions of DiPS using logistic regression (LR) and Support Vector Machine (SVM). These two methods are standard machine learning methods for classification. We used these two methods because they are well-established off-the-shelf methods. LR has strong interpretability which is important for model validation and SVM is served as a validation method to evaluate the robustness of this modeling approach to physical activity data. Tree-based models are not evaluated in this paper because their feature importance is difficult to interpret and different evaluation methods can lead to drastically different feature importance ranking.

### Logistic regression

Since our response variable (exercise relapse or not) is binary, our problem is a classification problem. Therefore, logistic regression is a favorable statistical approach because it has high interpretability and works well in practice. Logistic regression models the log-odds using an affine function.

The output of a prediction of the logistic regression model for a given set of estimated parameters is a numerical value indicating the likelihood of exercise relapse. In addition to prediction, a fitted logistic regression model can be valuable for interpretation, because we can identify the importance of features by considering their corresponding coefficients. We used the glm function with the binomial family in R [44] for LR implementation.

### SVM

Support Vector Machine (SVM) is a classification method that uses separating hyperplanes. SVM selects the hyperplane that gives the largest maximum distance to the training examples. We used the svm function in the e1071 package in R [44] for SVM implementation, and [45] provides more theoretical details about the SVM method.

### Train and test data

We trained the models using the preprocessed data collected in the first 15 weeks of the study. Then we used the trained model to predict exercise relapse for weeks 16–30. We compared our logistic regression (LR) model with 18 extracted features to the SVM model to demonstrate prediction accuracy.

### Simulation

In order to explore the potential benefit of using DiPS-based intervention, we used simulation to compare a DiPS-based intervention to a random intervention and a steps-based intervention, by assuming a simple dynamic step model with financial incentives. Our model assumes that for each participant, his/her steps for day ***n +*** **1** is determined as follows:

$$ {\boldsymbol{steps}}_{\boldsymbol{n}+\mathbf{1}}=\boldsymbol{\alpha} \bullet {\boldsymbol{steps}}_{\boldsymbol{n}}+\boldsymbol{C}+\boldsymbol{\epsilon} $$

where ***α*** is the correlation between steps for day n and steps for day n + 1, and *C* is a constant. Here, ***ϵ*** is a random variable that captures day-to-day fluctuations in physical activity, and we use the model that ***ϵ*** is a uniform distribution with range − *E* to *E* for a constant *E*. We further assume that ***steps***_**0**_ is the average steps during the run-in period. We used the mPED data to fit the above model for each participant so that the *i*-th participant has model parameters {***α***_***i***_, ***C***_***i***_, ***E***_***i***_}. We used these parameters when conducting our simulation.

In the simulation, we compare the number of adherent participants after a 3-month intervention period, where adherence is as defined in the methodology section: a participant is adherent if his/her steps in the latest week is greater or equal than his/her steps in the run-in period. We consider the following three policies for an intervention to increase adherence:

1. Random intervention: the probability of giving intervention for a given day and a given participant is ***p***^***∗***^.
2. Step based intervention: give intervention if observed daily step is below some threshold ***step***^***∗***^.
3. DiPS based intervention: give intervention if predicted DiPS score is below some threshold ***DiPS***^***∗***^.

For simplicity, we assume the intervention is a financial incentive (i.e., some dollar value for each intervention) and that giving the intervention will lead to an increase of 500 steps for that day. (The actual responsiveness to a fixed financial incentive will vary for each participant, and this sensitivity can be adaptively estimated for each participant using machine learning [16, 46]. We did not estimate this sensitivity for the simulation because financial incentives were not used in the mPED trial, and so the data needed to be able to estimate sensitivity is not available.) Therefore, after the parameter estimation phase, we use the resulting parameters to simulate data for a new study using one of the three intervention policies. Formally, we have:

$$ {\boldsymbol{step}}_{\boldsymbol{n}+\mathbf{1}}=\boldsymbol{\alpha} \bullet {\boldsymbol{step}}_{\boldsymbol{n}}+\boldsymbol{C}+\boldsymbol{\epsilon} +\mathbf{500}{\boldsymbol{u}}_{\boldsymbol{n}} $$

where ***u***_***n***_ follows one of the three intervention policies described above:

1. ***u***_***n***_ has a Bernoulli distribution with success probability ***p***^***∗***^.
2. $$ {\boldsymbol{u}}_{\boldsymbol{n}}=\left\{\begin{array}{c}\mathbf{1},\boldsymbol{if}\ {\boldsymbol{step}}_{\boldsymbol{n}-\mathbf{1}}<{\boldsymbol{step}}^{\ast}\\ {}\mathbf{0},\boldsymbol{otherwise}\end{array}\right. $$
3. $$ {\boldsymbol{u}}_{\boldsymbol{n}}=\left\{\begin{array}{c}\mathbf{1},\boldsymbol{if}\ {\boldsymbol{DiPS}}_{\boldsymbol{n}}<{\boldsymbol{DiPS}}^{\ast }\ \boldsymbol{and}\ \boldsymbol{n}\ \boldsymbol{\operatorname{mod}}\ \mathbf{7}\ \boldsymbol{is}\ \mathbf{1}\ \left(\boldsymbol{i}.\boldsymbol{e}.,\boldsymbol{first}\ \boldsymbol{day}\ \boldsymbol{of}\ \boldsymbol{week}\right)\\ {}\mathbf{0},\boldsymbol{otherwise}\end{array}\right. $$

For this simulation, DiPS is computed using the steps data and the week number since we do not observe the other features. Also, the first two policies are assessed daily, while the DiPS policy is assessed weekly and its intervention occurs only on the first day of the week when the predicted DiPS is smaller than the threshold. We select a sequence of values for ***p***^***∗***^***,step***^***∗***^***,DiPS***^***∗***^, compute spending for each scenario and compare adherence results under the three policies for the set of thresholds with comparable total spend.

## Results

**Fig. 2:**
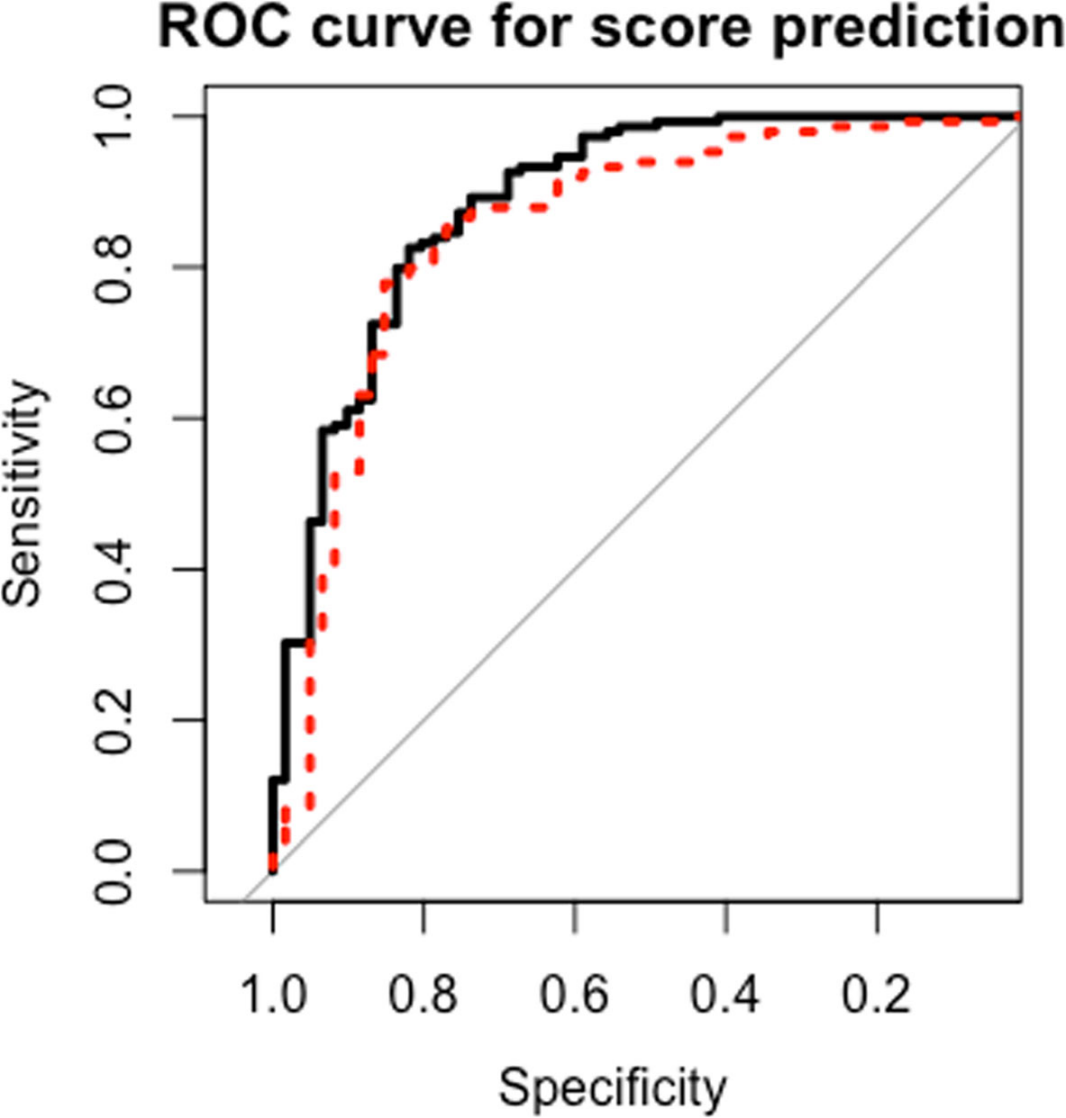
LR and SVM Prediction results. Caption: Receiver Operating Characteristics (ROC) curve of the predictions for week 20 using Augmented LR and SVM: black solid line is LR and red dash line is SVM.

We evaluated the performance of the models by comparing their Receiver Operating Characteristics (ROC) curve and Area Under Curve (AUC), where an AUC close to 1 indicates better performance of the classification task. Table 1 shows the AUC of the predictions for weeks 16–30 using the model trained by data from the first 15 weeks (including the 3 weeks of run-in and 12 weeks of intervention). Overall, the LR model has a higher average test AUC of 0.9016, and the SVM model has a slightly lower average test AUC of 0.8855. The high accuracy of both models indicates the robustness of the selected features in predicting DiPS. We show an example of the AUC curves of the two models in week 20 (the AUC curves of other weeks are similar) in Fig. 2 and observe that the optimal thresholds for the two models both have high accuracy (*>* 80%) and high specificity (*>* 80%).

**Table 1:** Test AUC for predicting weeks 16–30 using the fitted model

Week	16	17	18	19	20	21	22	23
LR	0.932	0.861	0.900	0.893	0.892	0.925	0.884	0.916
SVM	0.905	0.866	0.886	0.879	0.882	0.900	0.862	0.894
Week	24	25	26	27	28	29	30	Mean
LR	0.876	0.920	0.912	0.900	0.900	0.915	0.899	0.902
SVM	0.825	0.900	0.904	0.885	0.905	0.889	0.899	0.886

Table 2 displays the confusion matrix of the observed and predicted class for week 20 using the LR model with a threshold of 0.5 (this is an example threshold, and not necessarily the optimal). DiPS obtains an accuracy of 85% (179) and a specificity of 67% (141). The prediction accuracies in other weeks are comparable to week 20, confirming the overall robustness of the model. Note the above analysis was conducted on all participants, disregarding the randomization group. Therefore, we conducted additional analysis to evaluate whether the performance of the algorithm is consistent with different randomization groups. We conducted the ROC and AUC analysis for each of the individual groups on their test data from weeks 16–30 and present the results in Table 3. The outcome indicates that in the beginning weeks of the maintenance period, the test AUC of the CONTROL Group is lower than that of the other two groups. But toward later weeks, the test AUC of the CONTROL group increases. The PLUS group has the highest test AUC for all weeks during the maintenance period, except for week 20. The test AUC on the REGULAR group is between those of the CONTROL group and the PLUS group for 5 of the 20 weeks.

**Table 2:** Confusion matrix of the observed and predicted class for week 20 using the augmented Logistic Regression approach with a threshold of 0.5

		True Class		
		Relapse	Not Relapse	Total
Predicted class	Relapse	25% (53)	23% (49)	102
	Not Relapse	4% (8)	48% (100)	108
	Total	61	149	210

**Table 3:** Test AUC for predicting weeks 16-30 for each group using the fitted model

Week	CONTROL Group	REGULAR Group	PLUS Group
16	0.619	0.878	0.968
17	0.804	0.888	0.986
18	0.810	0.908	0.945
19	0.784	0.891	0.983
20	0.867	0.911	0.889
21	0.904	0.916	0.945
22	0.894	0.850	0.954
23	0.871	0.902	0.976
24	0.846	0.809	0.963
25	0.908	0.914	0.943
26	0.875	0.888	0.912
27	0.907	0.882	0.929
28	0.882	0.857	0.947
29	0.848	0.864	0.957
30	0.893	0.831	0.959

### Model interpretation

An advantage of using LR as the machine learning methods is its interpretability. For a fitted LR, the importance of each feature can be assessed using the coefficients. Table 4 shows the feature importance for the fitted LR model using data collected during the first 15 weeks. Week number is highly significant (*p*-value *<* 0.001), and the negative coefficients indicate that as the study progressed, participants were more likely to have exercise relapses. Initial steps, mean steps and last week steps are also all highly significant in predicting exercise relapse (*p*-value *<* 0.001). In addition to steps, physical activity intensity turns out to be a predicative feature. The positive coefficient of last week intensity indicates that if a participant was doing higher intensity physical activity, she was less likely to have an exercise relapse.

**Table 4:** Feature importance for the fitted augmented Logistic Regression model for week 20

Feature	Coefficient	*P*-value
Intercept	1.378	0.033
Week number	−0.122	*<* 0.001
Initial average daily steps	−0.001	*<* 0.001
Average daily steps	0.0008	*<* 0.001
Last week average daily steps	0.0004	*<* 0.001
Initial MVPR morning	−0.029	0.138
Initial MVPR afternoon	0.027	0.041
Initial MVPR evening	0.015	0.242
Average MVPR morning	0.067	0.02
Average MVPR afternoon	−0.037	0.103
Average MVPR evening	0.005	0.805
Last week MVPR morning	−0.032	0.058
Last week MVPR afternoon	0.001	0.920
Last week MVPR evening	−0.003	0.797
Initial MVPA intensity	−0.158	0.479
Average MVPA intensity	−0.354	0.280
Last week MVPA intensity	0.515	*<* 0.001
Average goal achieving percentage	0.161	0.849
Last week goal achieving percentage	0.294	0.340

### Simulation

**Fig. 3:**
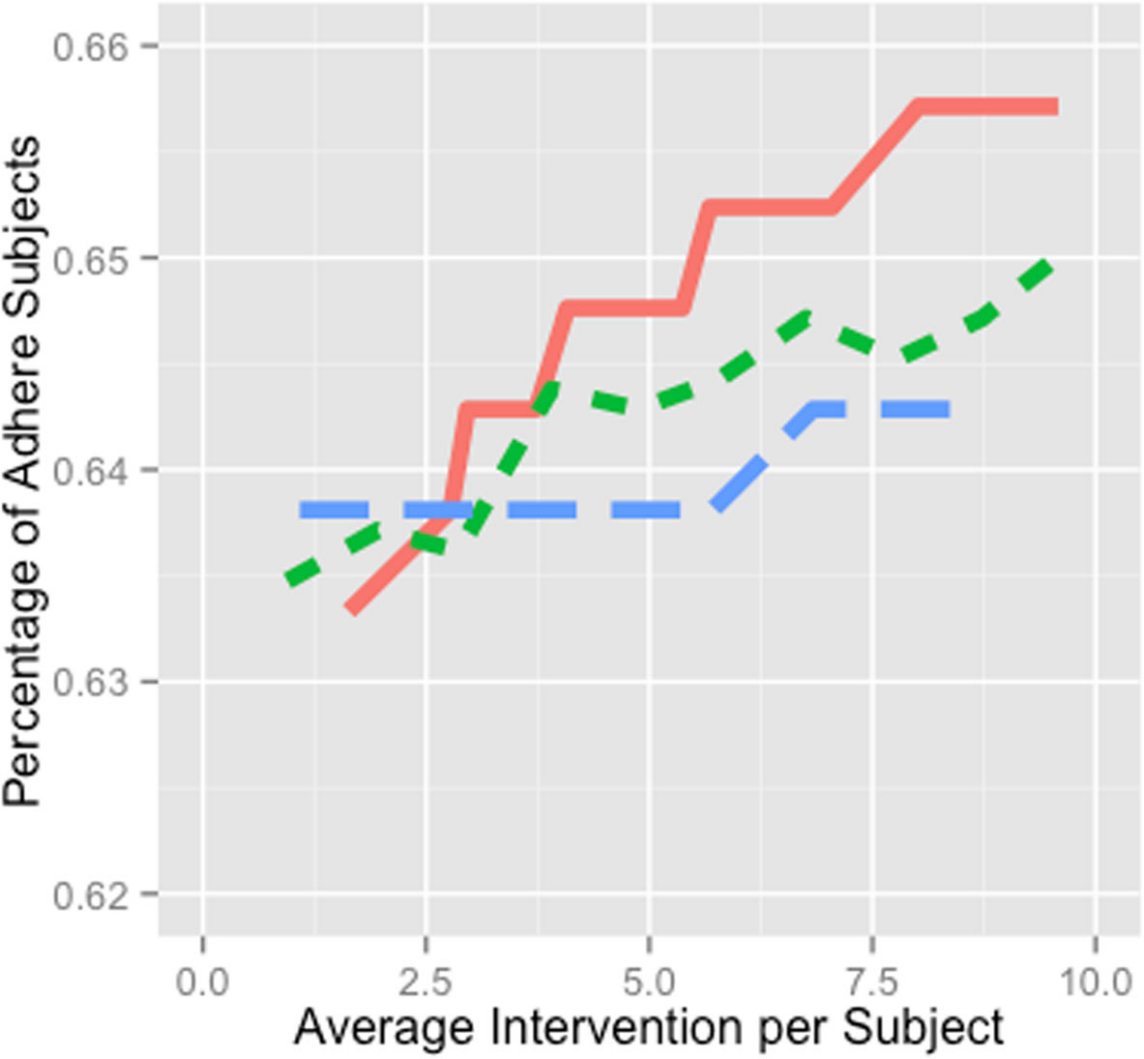
Simulation results for the three intervention policies. Caption: Simulation outcome of number of adhere participants after a 3-month trial with increasing spending under the three intervention policies: red solid line is DiPS based intervention; green shorter dash line is random intervention; blue dash line is step based intervention.

By assuming a simple dynamic step model with financial incentives, we used simulation to compare a DiPS-based intervention to a random intervention and a step-based intervention. Figure 3 shows the percentage of adherent participants versus number of interventions per participant for the three intervention policies. The steps policy (blue longer dash line) leads to the largest percentage of adherent participants when on average less than 2.6 interventions were delivered to each participant. As we increase the number of interventions, the DiPS policy (red solid line) leads to the largest percentage of adhere participants. The random policy (green short dash line) and steps policy (blue longer dash line) have lower performance, and the random policy appears to perform slightly better than the step-based policy.

## Discussion

### Accuracy and interpretation of DiPS

The results of our model suggest that DiPS has test accuracy around 80% and makes robust predictions across different weeks in this study sample. This suggests that DiPS could be a useful scoring for researchers and clinicians to tailor and adapt physical activity interventions to prevent exercise relapses. Note that this paper is less focused on finding the “best” ML method for this prediction problem, but rather validating that ML methods can be applied to the physical activity domain with high robustness. As a next step, we will evaluate/validate this framework against other ML methods, such as tree-based models.

The most predictive features coming out of the LR DiPS model are: week number, steps data (including initial average daily steps, average daily steps, last week steps), and physical activity intensity data. In contrast, preferred MVPA time in day was not significant. Furthermore, the coefficient of initial average daily steps is negative, indicating that participants with a higher physical activity level during the run-in period tended to have exercise relapse more often on average. This is intuitive since we define exercise relapse to be a comparison between current week steps and initial steps, thus higher initial steps means a more difficult baseline to beat. Furthermore, last week daily steps and average daily steps have positive coefficients, indicating that participants who were more active in the last week and over the entire study period were less likely to have exercise relapses.

### Efficient resource allocation using DiPS

The potential of DiPS to provide real-time feedback for generating just-in-time interventions for individuals likely to have an exercise relapse was explored through a simulation that compared the cost-effectiveness of different policies to allocate financial incentives to encourage selected individuals to increase their physical activity (Fig. 3).

Simulation outcome indicates that a DiPS-based intervention is more effective in enhancing adherence, compared to step-based and random interventions. Real-time interventions that improve adherence through mobile devices have not yet been implemented in physical activity intervention studies. Cadmus-Bertram conducted a study to track adherence using Fitbit, but no intervention was given in accordance to the collected data [46]. Other studies introduced human moderation to monitor adherence, and then schedule in-person sessions or intervention calls to improve adherence [47, 48]. However, it is important to note that the simulation makes many assumptions and is conducted to explore the benefit that could potentially result, rather than to validate the model. Thus an empirical study is warranted to confirm the simulation findings.

### Incorporation of DiPS into physical activity promotion programs

DiPS can be potentially incorporated into mobile technology based physical activity promotion programs that collect real-time activity data. With the recent rapid development of motion sensors and wearable devices, prediction models, such as DiPS, using real-time activity data, will allow researchers and clinicians to provide automatically generate individualized, just-in-time interventions. For example, intervention messages can be delivered through push notifications for app-based programs or through text messages. Such interventions can be triggered automatically when a low DiPS is predicted. The content of such interventions can provide an interactive dialog to identify the reason for relapse and provide personalized suggestions. In addition, DiPS can also assist in adjusting automated, personalized goals where future step goals are reduced for those who are ready to experience exercise relapse. In our recent trials, like other trials, we found that personalized automated personalized goal setting is more effective than standard goal setting [35, 49–53]. Thus, DiPS has great potential. However, several limitations need to be acknowledged. First, the trial data used in this paper were collected from only physically inactive female adults. Thus, DiPS scoring will need to be tested and validated in other populations, such as male adults and children. Second, since we did not have objectively measured physical activity data beyond 9 months, prediction of DiPS scoring beyond this time period might be lower than our findings in this study. Therefore, the efficacy of DiPS scoring for physical activity intervention needs to be tested in a full-scale RCT in the near future.

## Conclusion

DiPS is a machine learning-based score that uses logistic regression or SVM on objectively measured step and goal data, and it was able to accurately predict exercise relapse with a sensitivity of 85% and a specificity of 67%. In addition, simulation results suggest the potential benefit of DiPS as a score to allocate resources in order to hopefully provide more cost-effective interventions for increasing adherence. However, DiPS will need to be validated in larger and different populations, and its efficacy will need to be examined in a full-scale RCT in the near future.

## Data Availability

The datasets used for this paper are available from the corresponding author on reasonable request.

## Abbreviations

AUC: Area Under Curve
DiPS: Discontinuation Prediction Score
EWS: Early Warning Score
LR: Logistic Regression
METs: Metabolic Equivalents
mPED: Mobile Phone-Based Physical Activity Education
MVPA: Moderate to vigorous intensity physical activity
RCT: Randomized controlled trial
ROC: Receiver Operating Characteristics
SVM: Support Vector Machine
